# Supplementary figures and images for: Multiscale effects of excitatory-inhibitory homeostasis in lesioned cortical networks: A computational study
Source: PLoS Comput Biol. 2023 Jul 7;19(7):e1011279. doi: 10.1371/journal.pcbi.1011279 (PMC10355437; doi:10.1371/journal.pcbi.1011279)

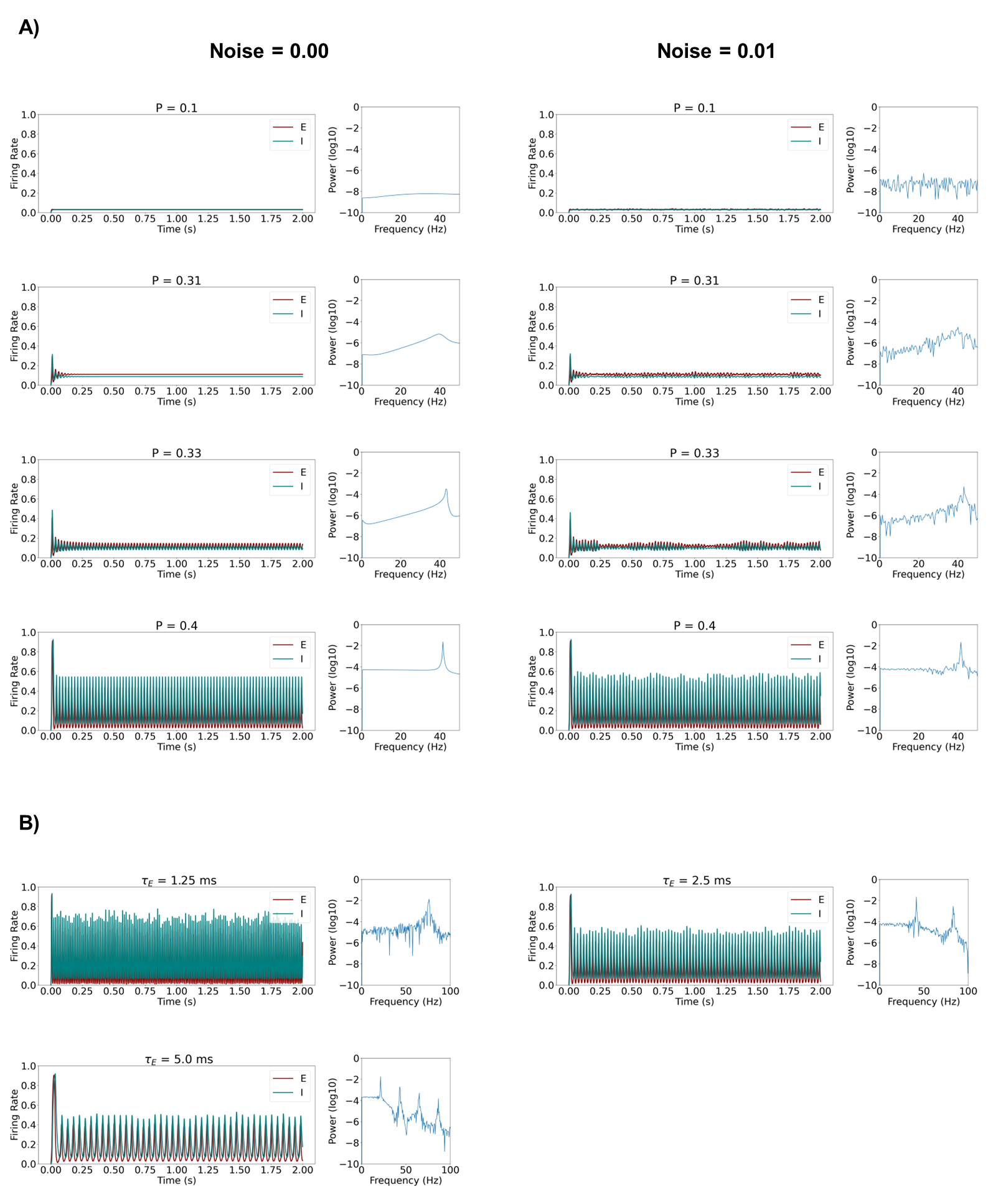

Supplement: S1 Fig — A) Impact of changing the parameter P which controls the intrinsic excitability of the Wilson Cowan node, on node activity and power spectrum. On the left side, we show results for models without noise and, on the right side, we show results of nodes with gaussian noise with 0.01 standard deviation. Note that, in our model, uncoupled nodes go from a state of low activity to a limit cycle (by increasing P, showing the behavior of a Hopf bifurcation For the chosen population time constants τE = 2.5ms τI = 5.0ms the Wilson Cowan model displays oscillations at 40 Hz. B) Impact of changing population time constants on the oscillatory dynamics of uncoupled noisy Wilson Cowan nodes (Gaussian noise, 0.01 standard deviation) For all shown plots, τI = 2τE It can be observed that the intrinsic frequency of oscillation of the Wilson Cowan nodes is changed by varying the time constants of the excitatory and inhibitory populations. (TIF) [file pcbi.1011279.s001.tif]

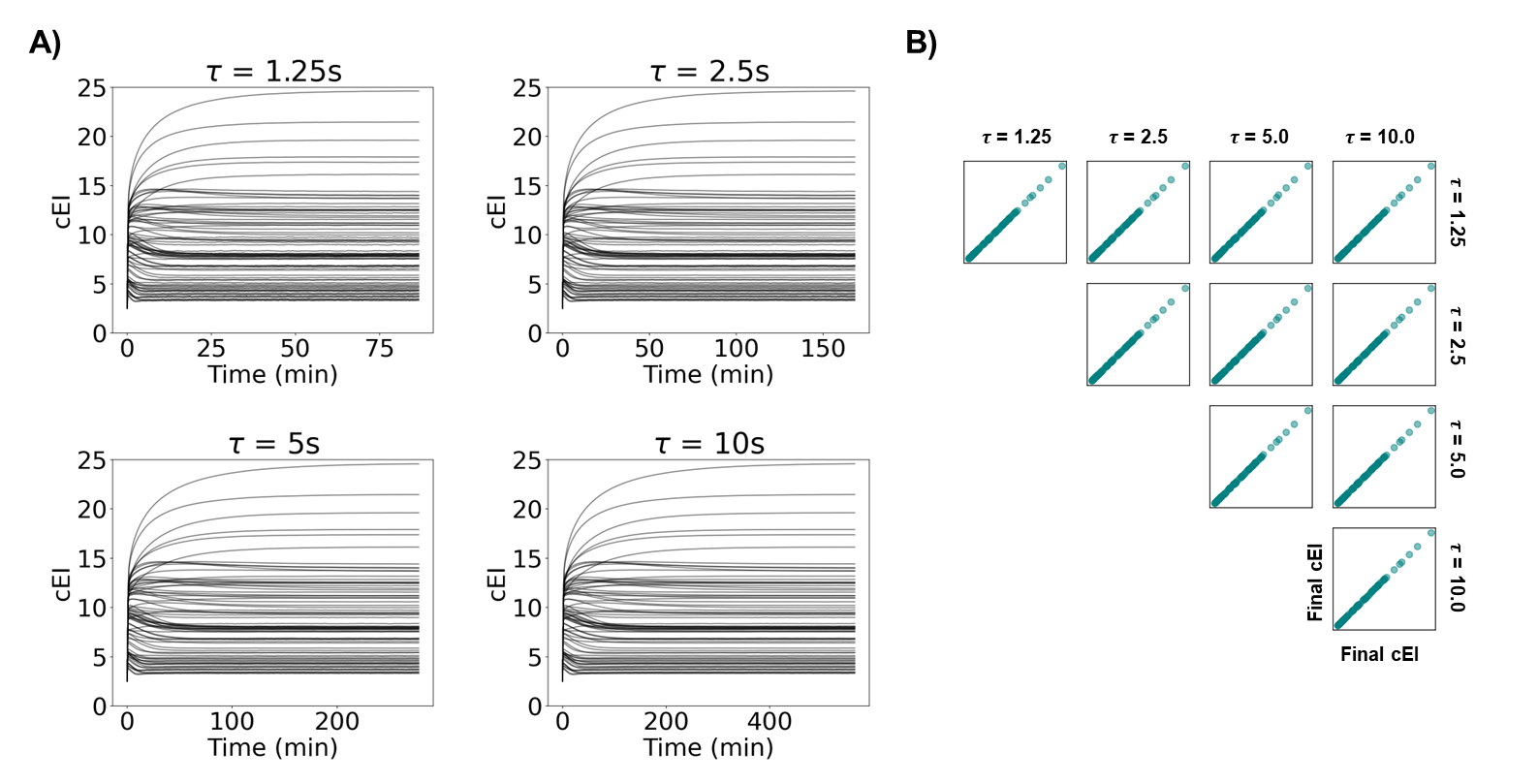

Supplement: S2 Fig — A) Variation in time in local inhibitory weights for all 78 nodes in the model, under different time constants of homeostatic plasticity, for the following combination of free parameters C = 4.07 ρ = 0.2 md = 4ms. Note that while cEI values take longer to reach a steady state for slower time constants, the final steady state values are virtually the same. B) Scatter plots of steady state c EI values for each homeostatic time constant against each other Note that values are virtually the same, showing that, as long as the homeostatic time constant is sufficiently slow to be decoupled from local node dynamics, it can be arbitrarily fast without affecting the steady state of the system. (TIF) [file pcbi.1011279.s002.tif]

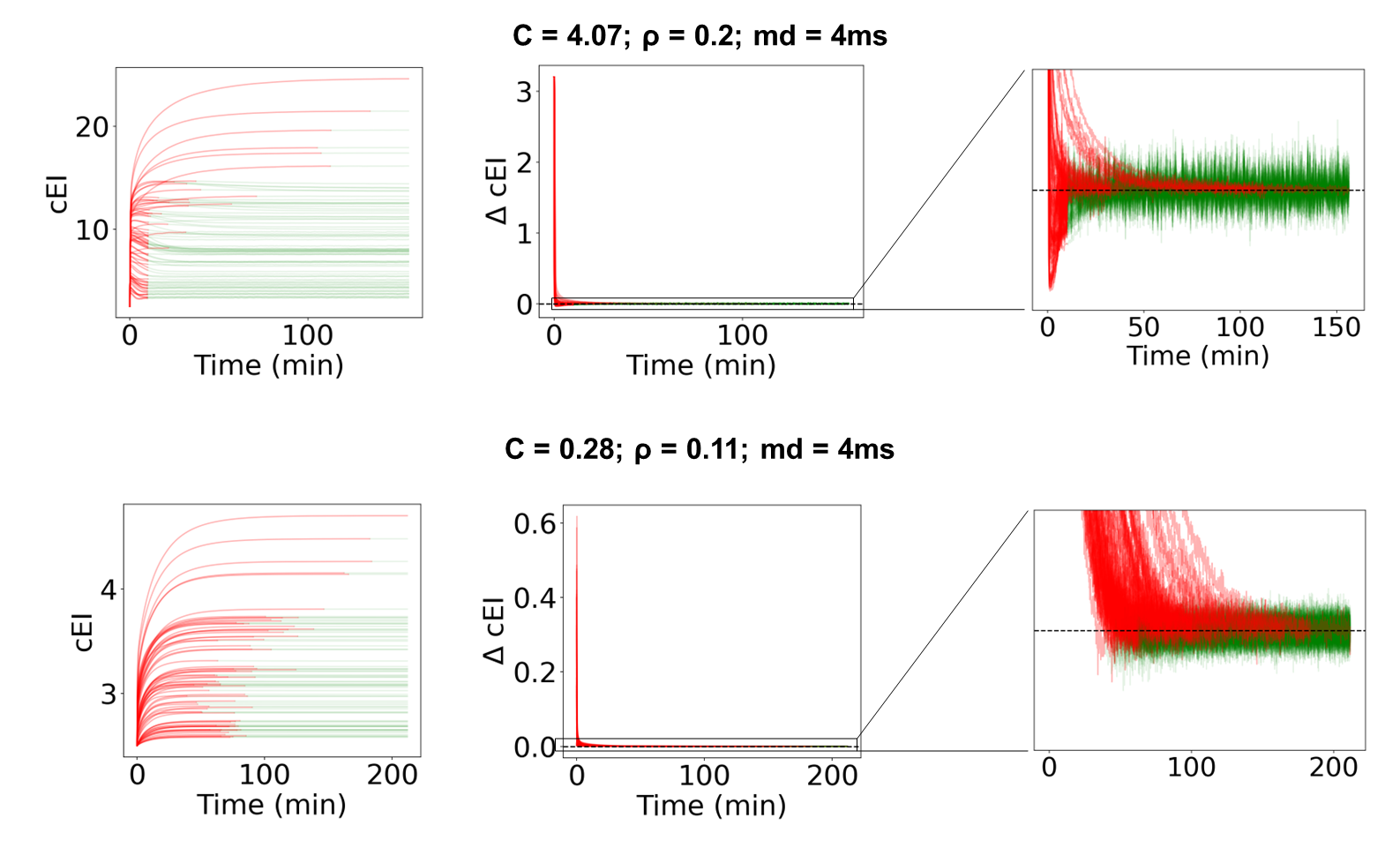

Supplement: S3 Fig — (TIF) [file pcbi.1011279.s003.tif]

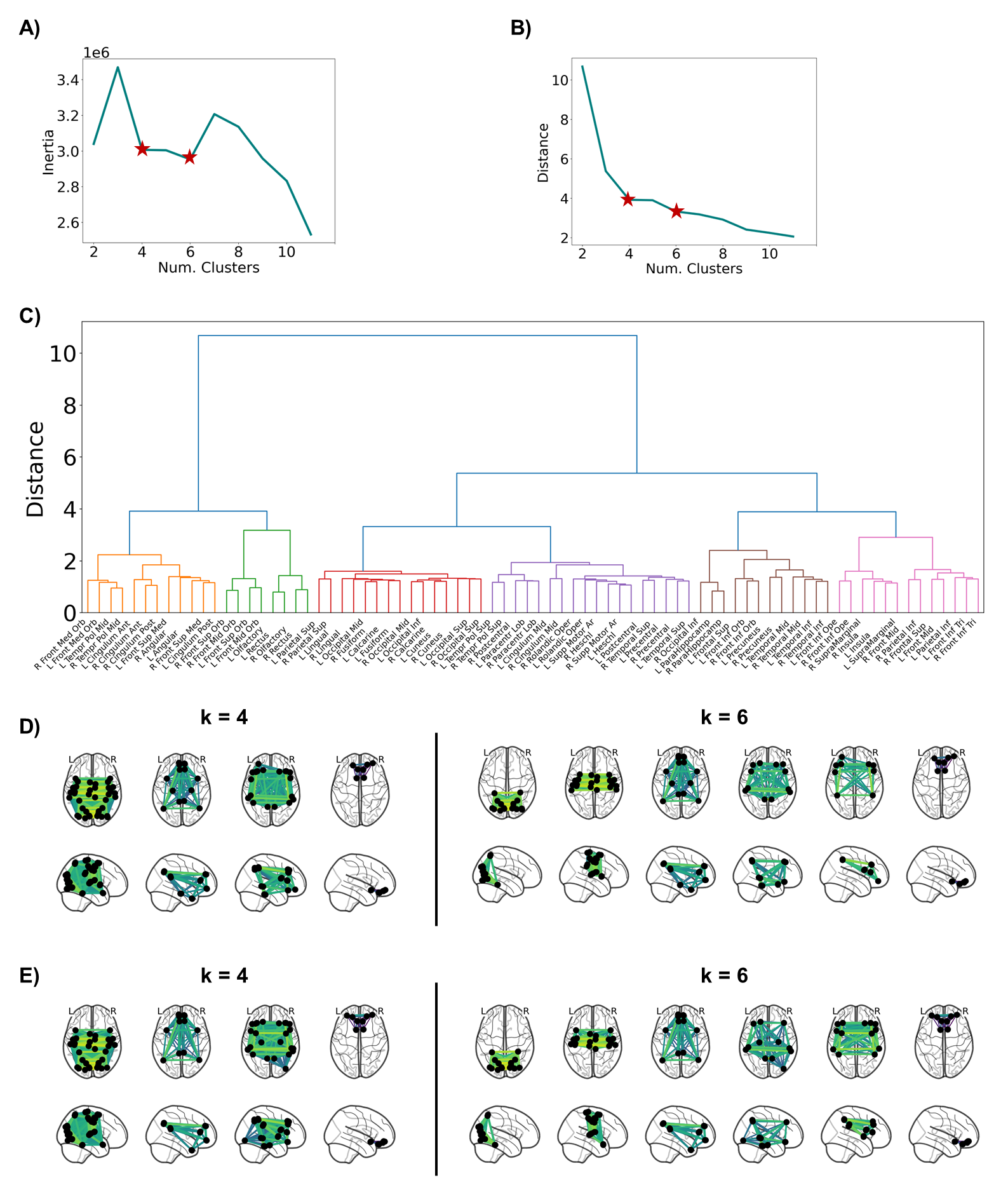

Supplement: S4 Fig — A) Resulting cluster inertia from applying the k-means algorithm described in the methods to empirical averaged functional connectivity from healthy subjects, with different numbers of clusters. Stars indicate potential ’elbows’ in the cluster analysis, i.e. local minima or points with an inflection in inertia relative to the number of clusters. Inertia was calculated using the scikit learn module in Python. B) Resulting cluster distance from hierarchical clustering to averaged functional connectivity from healthy subjects, with different numbers of clusters. Stars indicate potential ’elbows’ in the cluster analysis, i.e. local minima or points with an inflection in distance relative to the number of clusters. Hierarchical clustering was computed using the scikit learn module in Python. C) Dendrogram of averaged functional connectivity from healthy subjects. Colors represent 6 different clusters. D) Functional networks resulting from the application of the k means clustering algorithm to empirical data with 4 and 6 clusters. Note that the resulting networks for k = 6 can be equated to known resting state networks (e.g. visual (first) somatomotor (second) and default mode network (third)). E) Functional networks resulting from the application of hierarchical clustering to empirical data with 4 and 6 clusters. Note that the resulting networks for both k = 4 and k = 6 are reasonably similar to the ones in D), with known resting state networks emerging when k = 6. (TIF) [file pcbi.1011279.s004.tif]

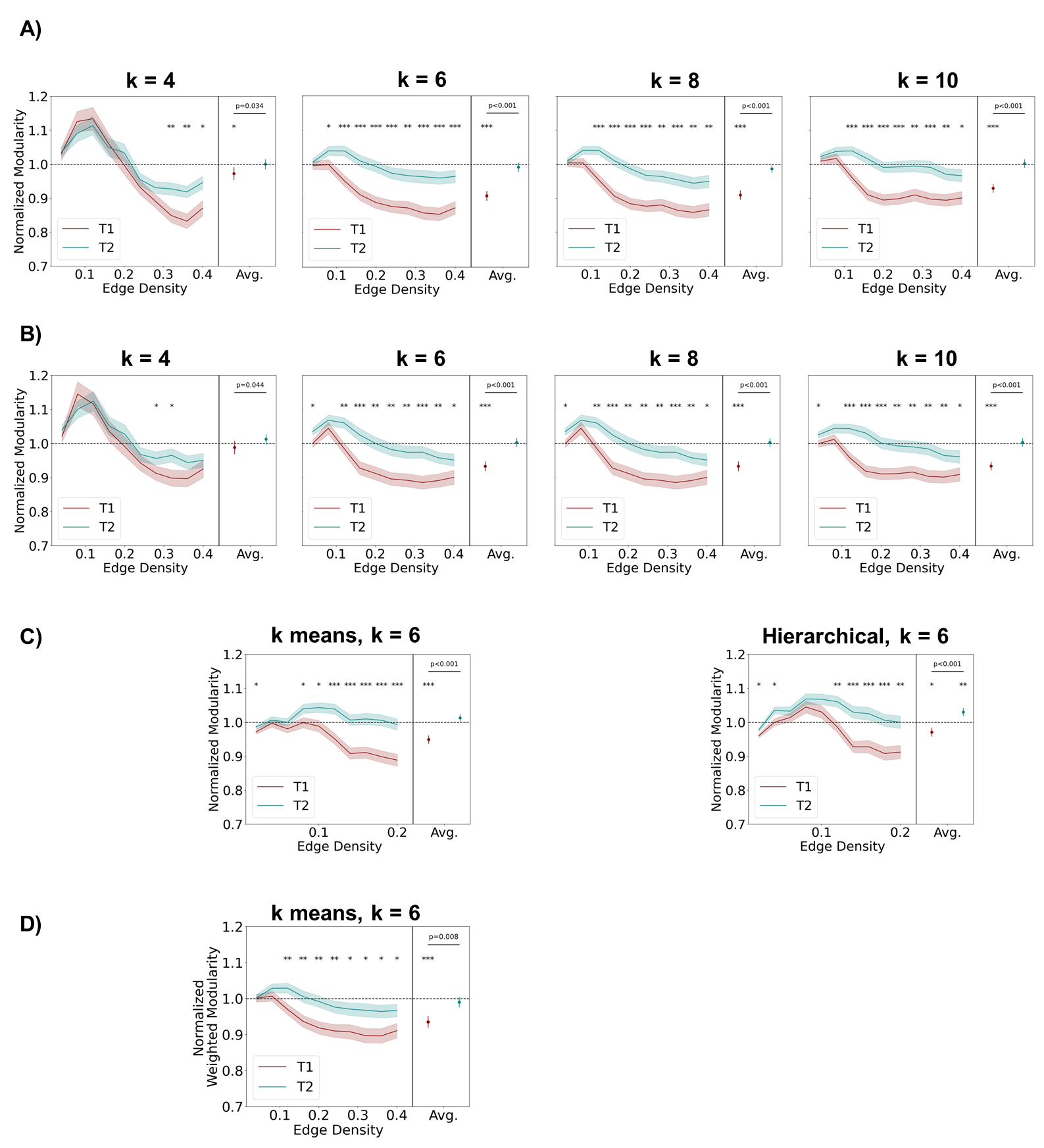

Supplement: S5 Fig — A) Normalized modularity at T1 (acute post-lesion) and T2 (chronic post-lesion) for different results of k-means clustering. Each plot represents modularity analysis using as modules the result of k-means with the number of clusters ranging from 4 (left) to 10 (right). In each plot, we present results across a range of density thresholds and the average across density thresholds. Across density thresholds, asterisks represent the level of significance of a Mann-Whitney U-test. For the average across density thresholds, asterisks represent the level of significance of a Wilcoxon ranked sum test against baseline (norm. mod. = 1). * p<0.05, ** p<0.01, *** p<0.001. B) Same as A), but for modules derived from hierarchical clustering. C) Normalized modularity at T1 (acute post-lesion) and T2 (chronic post-lesion) for edge-density thresholds ranging between 0.02 and 0.2, with 6 modules derived from k-means (Left) or hierarchical clustering (Right). In each plot, we present results across the range of density thresholds and the average across density thresholds. Across density thresholds, asterisks represent the level of significance of a Mann-Whitney U-test. For the average across density thresholds, asterisks represent the level of significance of a Wilcoxon ranked sum test against baseline (norm. mod. = 1). * p<0.05, ** p<0.01, *** p<0.001. D) Normalized modularity at T1 (acute post-lesion) and T2 (chronic post-lesion) using a weighted modularity algorithm. We present results across a range of density thresholds and the average across density thresholds. Across density thresholds, asterisks represent the level of significance of a Mann-Whitney U-test. For the average across density thresholds, asterisks represent the level of significance of a Wilcoxon ranked sum test against baseline (norm. mod. = 1). * p<0.05, ** p<0.01, *** p<0.001. (TIF) [file pcbi.1011279.s005.tif]

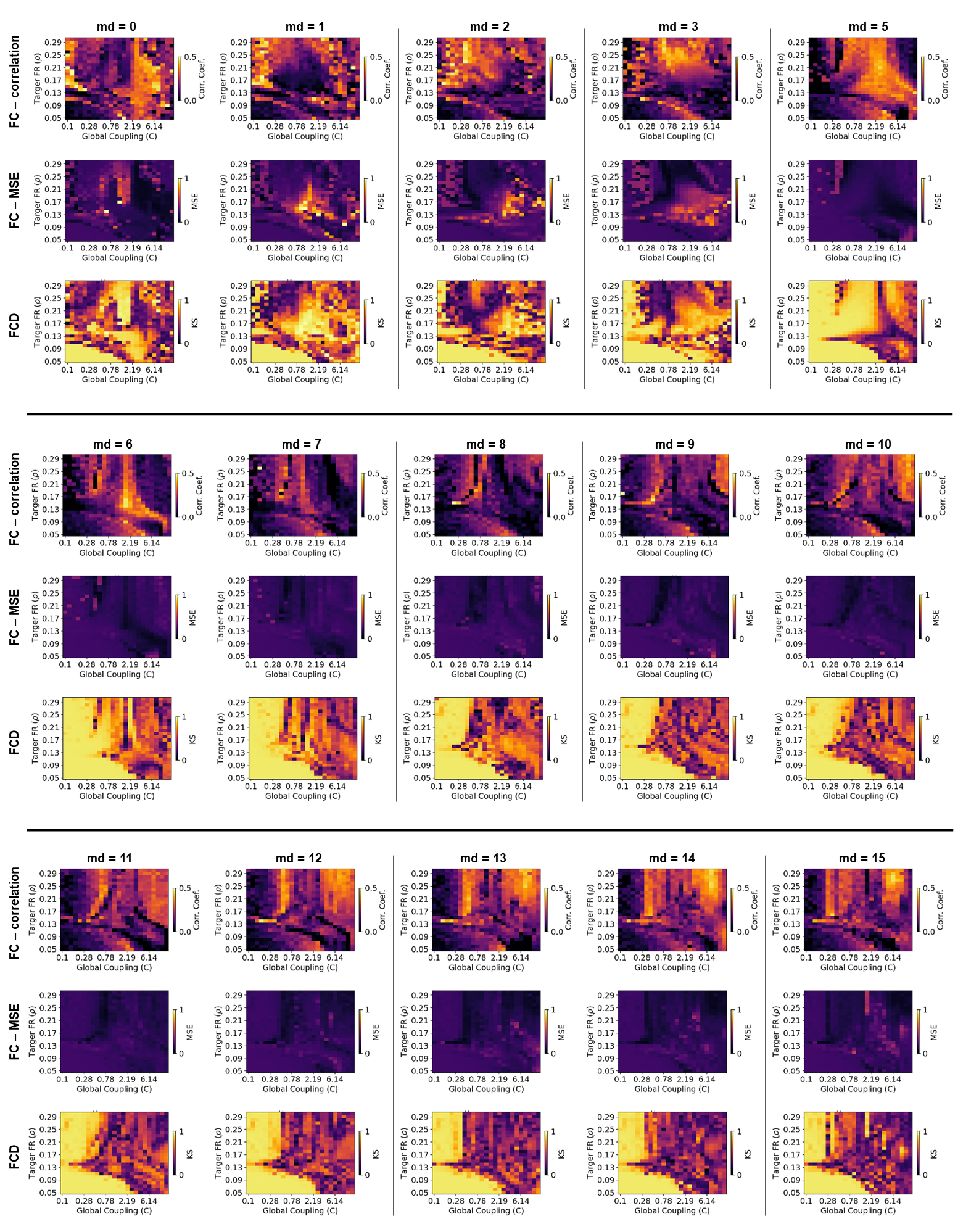

Supplement: S6 Fig — Model fit over full parameter space. Each column of three plots represents the results of a grid search over the parameters of global coupling (C) and target firing rate (FR) (ρ), for a specific mean delay between 0 and 15 ms. In each column, model performance is shown according to the following metrics: (Top) Pearson’s correlation between the upper triangle of simulated and empirical FC matrices, (Middle) mean squared error (MSE) between simulated and empirical FC matrices and (Bottom) Kolmogorov-Smirnoff (KS) distance between the distribution of values in simulated and empirical FCD matrices. (TIF) [file pcbi.1011279.s006.tif]

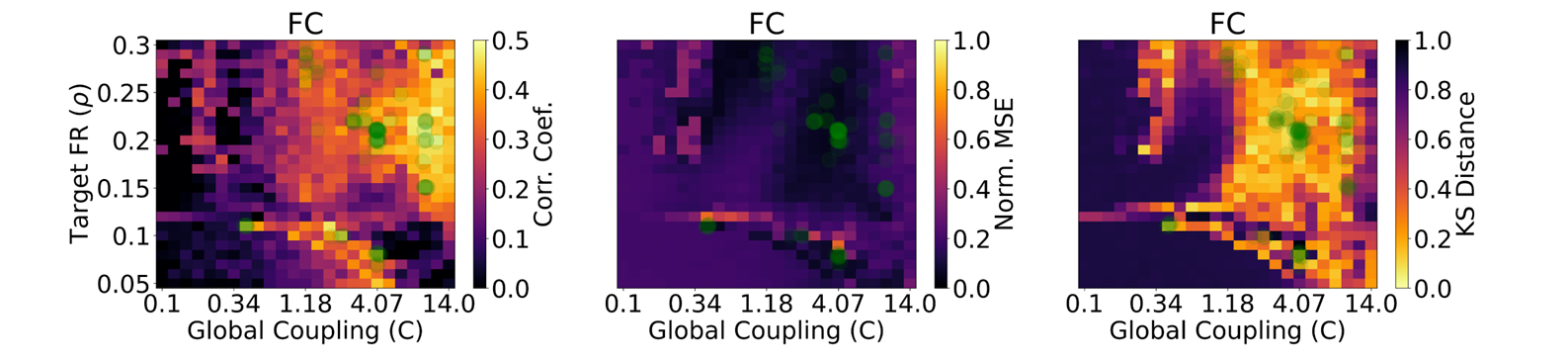

Supplement: S7 Fig — The colormaps represent the model fit over full parameter space, obtained by comparing the average FC and aggregated FCD distribution from empirical data with model results. Model performance is evaluated according to the following metrics: (Left) Pearson’s correlation between the upper triangle of simulated and empirical FC matrices, (Middle) mean squared error (MSE) between simulated and empirical FC matrices and (Right) Kolmogorov-Smirnoff (KS) distance between the distribution of values in simulated and empirical FCD matrices. The green dots represent the optimal point obtained from the same procedure when applied to subject-specific FC matrices and FCD distributions. Note the higher concentration of optimal points around the parameter region used as the optimal working point for our simulations (C = 4.07, ρ = 0.2). (TIF) [file pcbi.1011279.s007.tif]

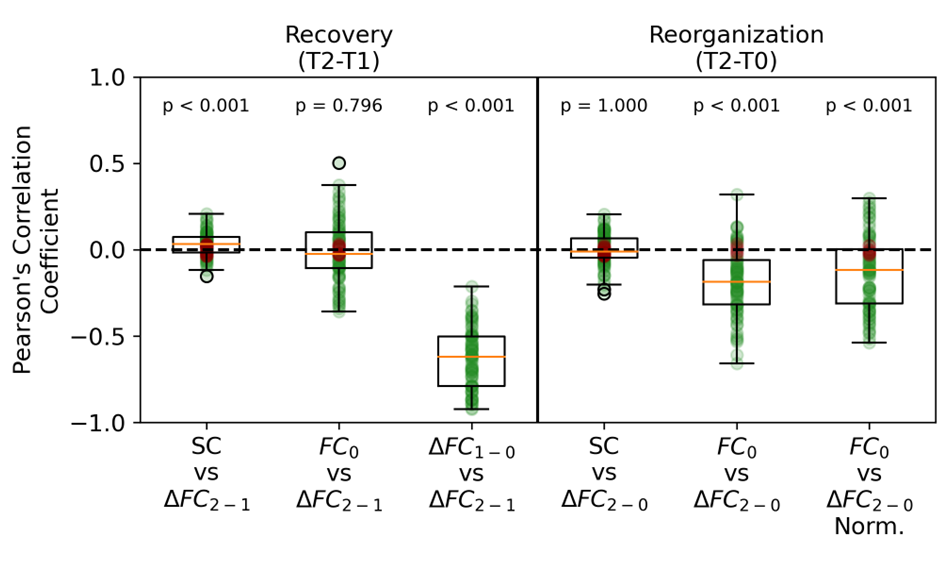

Supplement: S8 Fig — Correlation between recovery and reorganization of FC across lesions. We classify differences between T1 and T2 as recovery, since they are related to the recovery of acute FC deficits and differences between T2 and T0 as reorganization, since they quantify the reorganization of FC connections that is required for recovery. From left to right, we show the correlation across lesions between SC and ΔFC(T2-T1), FC(0) and ΔFC(T2-T1), ΔFC(T2-T1) and ΔFC(T1-T0), SC and ΔFC(T2-T0), FC(T0) and ΔFC(T2-T0), FC(T0) and ΔFC(T2-T0) normalized to the maximum possible change, i.e. (FC(T2)-FC(T0))/(1 –FC(T0)). Dots represent the correlation between each of the combinations of measures on an individual lesion level. Dot color indicates the significance of the correlation, with green representing FDR corrected p-value < 0.05. Boxplots represent the distribution across lesions. P-values above each boxplot represent the result of a Wilcoxon ranked-sum test, adjusted for multiple comparisons with FDR correction. Note that recovery is strongly negatively correlated with acute deficit in FC (third boxplot), indicating that deficits are being appropriately corrected from T1 to T2. In addition, reorganization is generally negatively correlated with healthy FC (T0) (fifth boxplot), meaning that reorganization is underlined by increases in the weakest healthy functional connections, which can be conceptualized as the formation of new functional connections. The significance of these results is maintained when normalizing for maximum possible increase in FC (sixth boxplot). (TIF) [file pcbi.1011279.s008.tif]

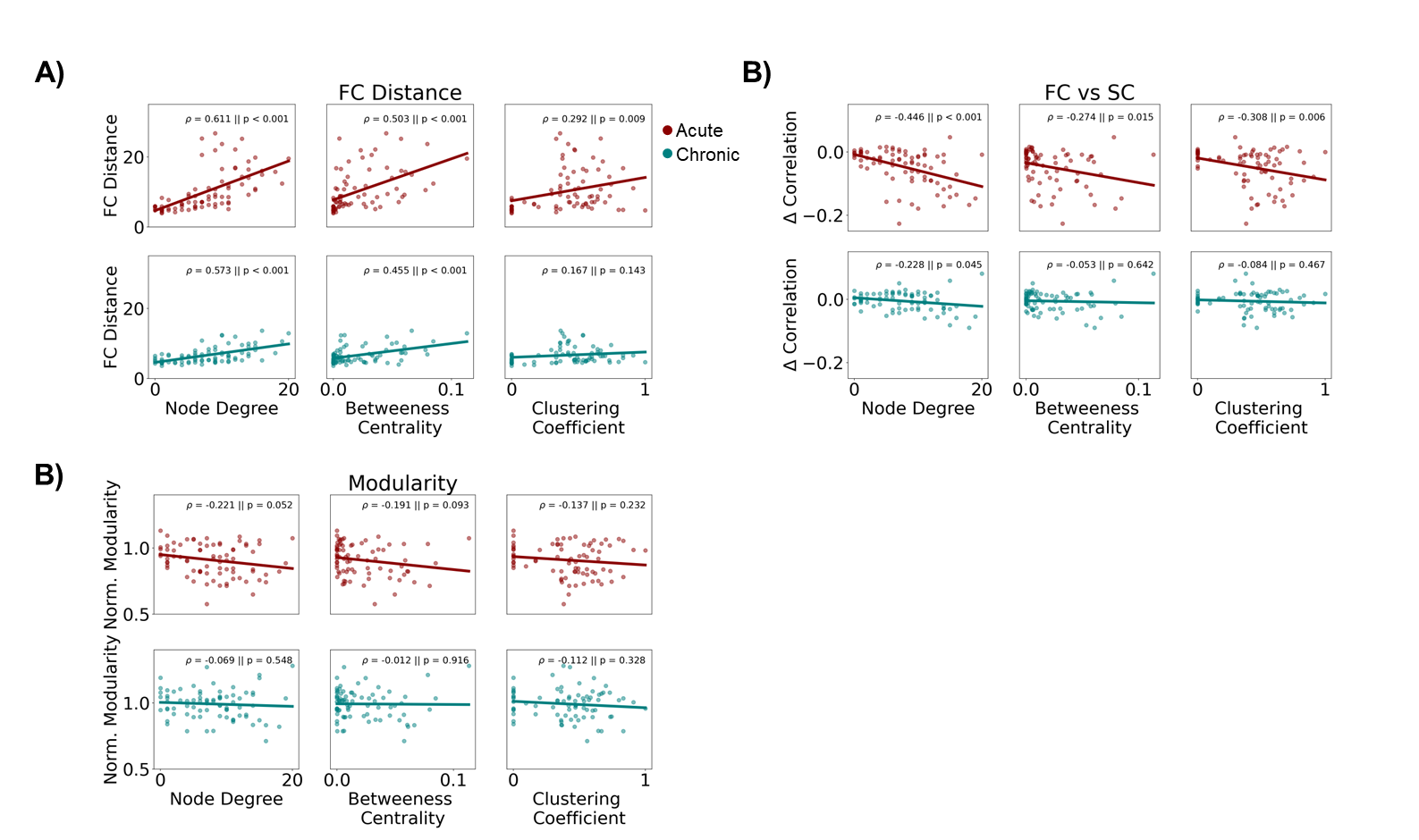

Supplement: S9 Fig — A) Distance from baseline FC matrices at T1 (acute post-lesion) and T2 (chronic post-lesion) against node degree, betweenness centrality and clustering coefficient of lesioned nodes. All graph theoretical measures of lesioned nodes used in the plots were calculated using the networkx module in Python, after transforming the SC matrix into an undirected unweighted graph by thresholding the 10% strongest structural connections. B) Same as A), for the difference in correlation between structural and functional connectivity at T1 and T2, compared to baseline. C) Same as A), for normalized modularity at T1 (acute post-lesion) and T2 (chronic post-lesion). Normalization was calculated using the value at T0 as the baseline. (TIF) [file pcbi.1011279.s009.tif]

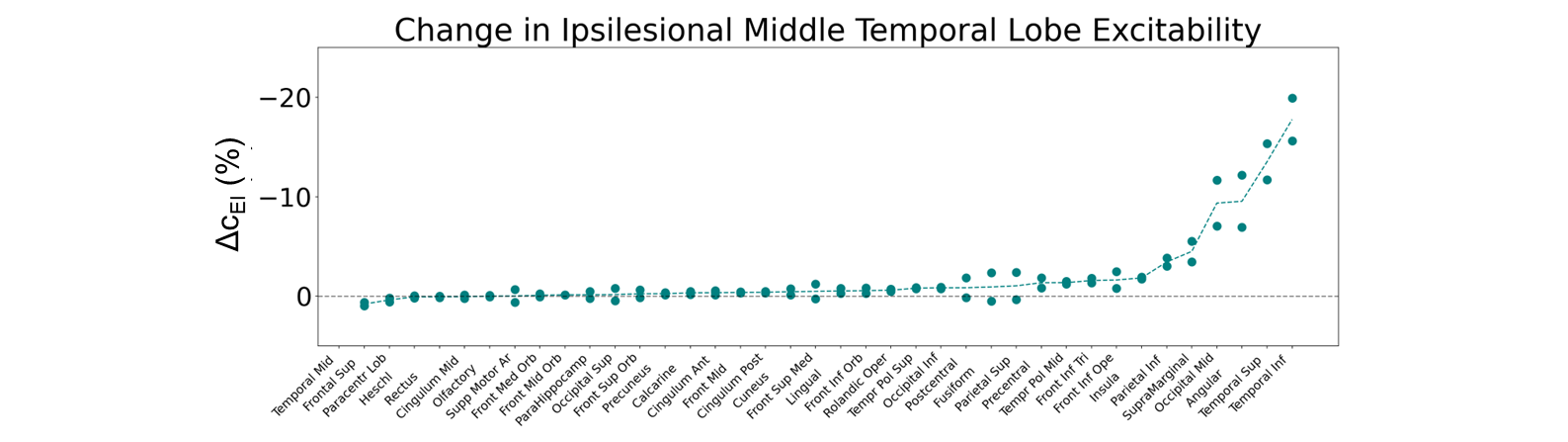

Supplement: S10 Fig — Variation, between T0 and T2, in cEI weight of the middle temporal cortex after lesion in the same hemisphere. Points represent results for left and right lesions in the respective areas and the dashed line represents the average between these two values. Areas are ordered according to the average effect on middle temporal cortex excitability. (TIF) [file pcbi.1011279.s010.tif]

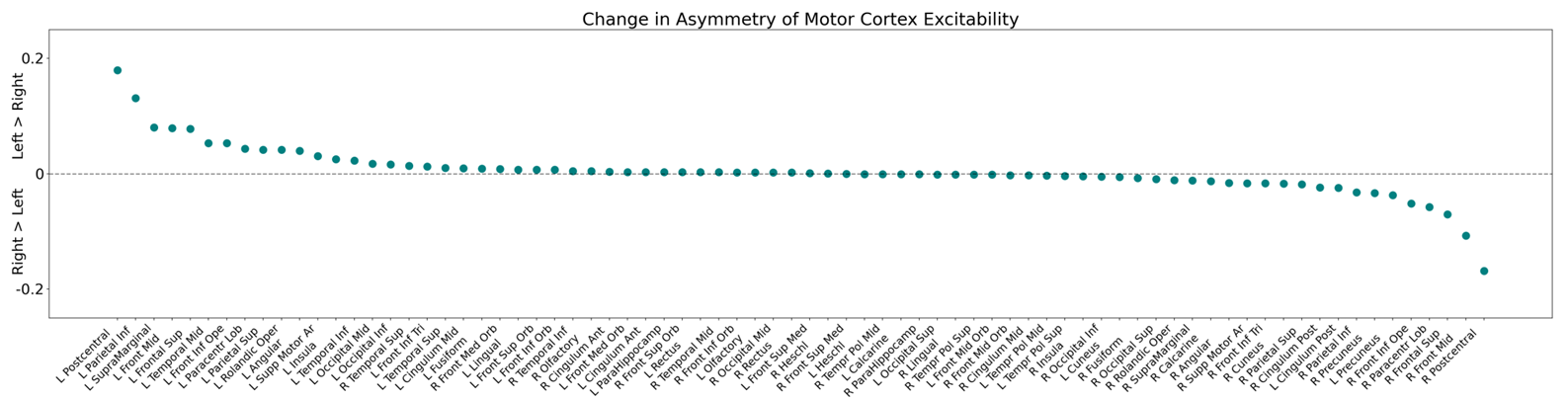

Supplement: S11 Fig — Variation, between T0 and T2, in motor cortex (precentral gyrus) excitability asymmetry across all lesions. Positive values indicate that the left motor cortex experienced a stronger increase in excitability when compared to its right counterpart, while negative values indicate the opposite variation. Areas are ordered according to lesion effects in this asymmetry. (TIF) [file pcbi.1011279.s011.tif]

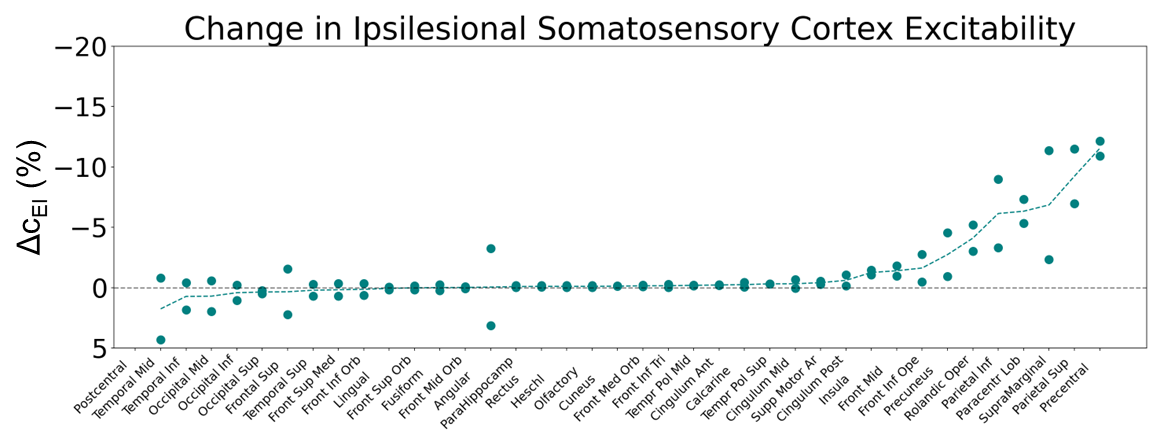

Supplement: S12 Fig — Variation, between T0 and T2, in cEI weight of the somatosensory cortex (postcentral gyrus) after lesion in the same hemisphere. Points represent results for left and right lesions in the respective areas and the dashed line represents the average between these two values. Areas are ordered according to the average effect on somatosensory cortex excitability. (TIF) [file pcbi.1011279.s012.tif]

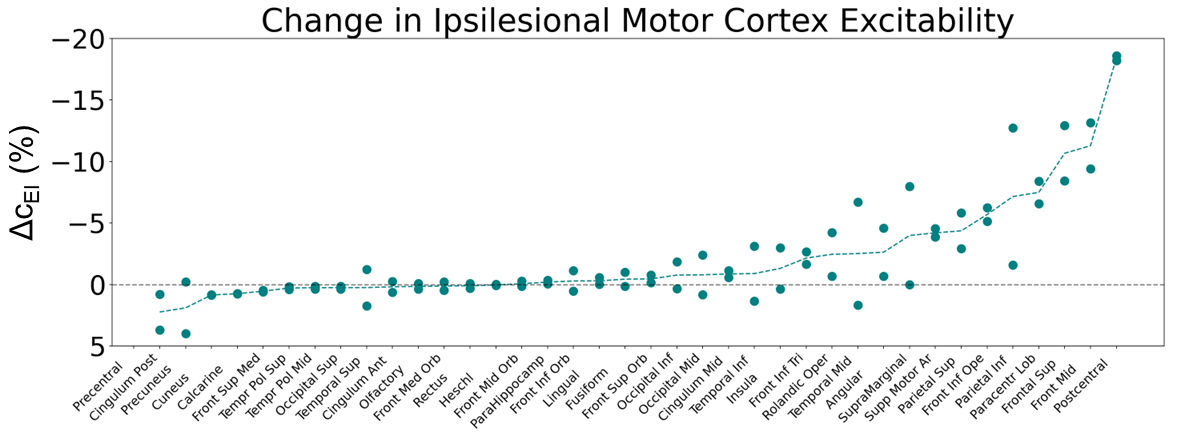

Supplement: S13 Fig — Variation, between T0 and T2, in cEI weight of the motor cortex (precentral gyrus) after lesion in the same hemisphere. Points represent results for left and right lesions in the respective areas and the dashed line represents the average between these two values. Areas are ordered according to the average effect on motor cortex excitability. (TIF) [file pcbi.1011279.s013.tif]

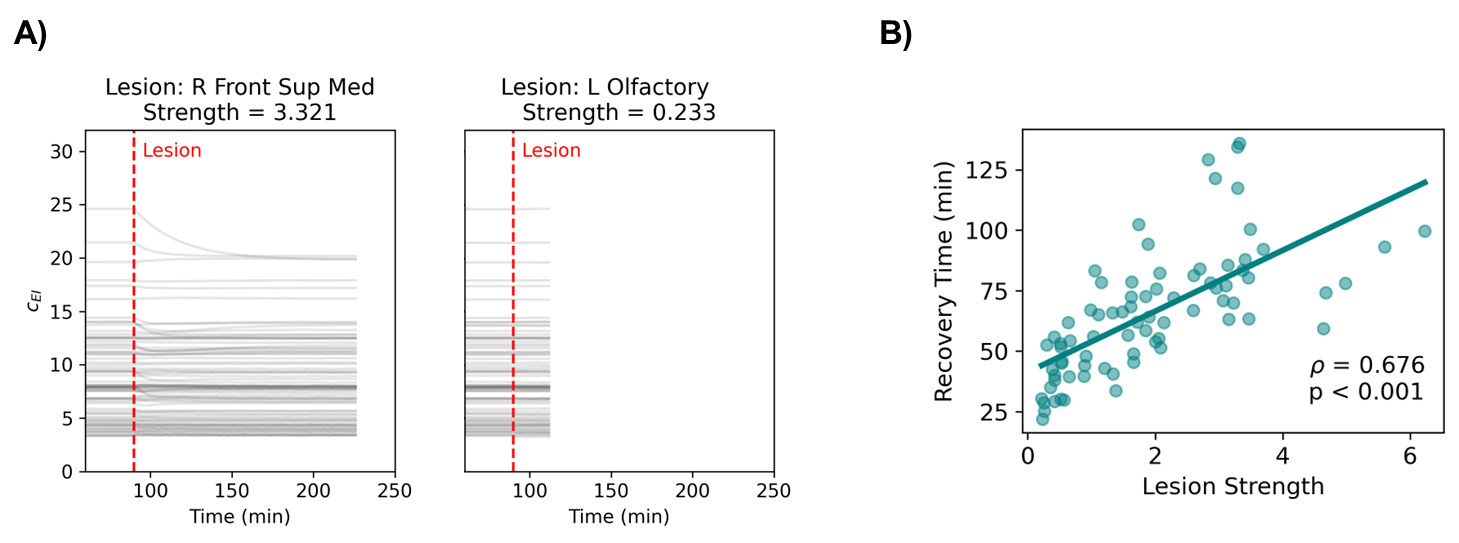

Supplement: S14 Fig — A) Example of the evolution of local inhibitory weights (cEI) after lesion in a high strength node (Superior frontal gyrus, medial orbital) and a low strength node (Left Olfactory Cortex). B) Correlation between lesion strength and time required for cEI stabilization after lesion. Lesions in nodes with strong structural connections require longer adaptation times for the restoration of local E-I balance. (TIF) [file pcbi.1011279.s014.tif]
