# Supplementary material for: Multiscale effects of excitatory-inhibitory homeostasis in lesioned cortical networks: A computational study
Source: PLoS Comput Biol. 2023 Jul 7;19(7):e1011279. doi: 10.1371/journal.pcbi.1011279 (PMC10355437; doi:10.1371/journal.pcbi.1011279)
Supplement: S1 Text — (PDF) [file pcbi.1011279.s015.pdf]

Every 10 seconds, a vector keeping a down-sampled version of c_{EI} in the last 10 minutes is created for every node as follows:

$$c_{EI_{vec}}(t) = [c_{EI}(t - T_{window}), c_{EI}(t - T_{window} + 10s), \quad (\dots), \quad c_{EI}(t - 10s), c_{EI}(t)], \quad T_{window} = 600s$$

Then, the following test condition is applied, using $dc_{EI_{vec}}$, the difference between consecutive elements in $c_{EI_{vec}}$

$$|mean(dc_{EI,vec})| < \frac{std(dc_{EI_{vec}})}{\sqrt{N}}$$

When this condition is satisfied in a specific node for the first time during a simulation, we consider that node to have reached a steady state in terms of c_{EI} weight. Shortly, if the absolute mean change of c_{EI} for that specific node in the last 10 minutes is smaller than the standard error of the mean in the same period, the value is considered stable. Since the rate of variation of c_{EI} decreases until the local firing rate is brought close to the target firing rate, $|mean(dc_{EI,vec})|$ will decrease until it approaches 0. However, one must account for the stochasticity of the system, and that is why we compare the mean variation with its respective standard error. Therefore, we effectively detect when the tendency of variation caused by homeostatic plasticity trying to restore EI balance is smaller than changes caused by the inherent stochasticity of the model.

When a steady state has been reached in all nodes or 500 minutes have passed, plasticity is disabled and activity is recorded from the model.
