# Supplementary material for: Multiscale effects of excitatory-inhibitory homeostasis in lesioned cortical networks: A computational study
Source: PLoS Comput Biol. 2023 Jul 7;19(7):e1011279. doi: 10.1371/journal.pcbi.1011279 (PMC10355437; doi:10.1371/journal.pcbi.1011279)
Supplement: Attachment — Submitted filename: response_letter.pdf [file pcbi.1011279.s016.pdf]

Dear Editors,

We thank you for the opportunity to resubmit a revised version of the manuscript for publication in the PLOS Computational Biology journal. We greatly appreciate the effort and time that you and the reviewers have devoted to reading our manuscript and providing insightful feedback. Therefore, we have been able to update our manuscript to include most of the revisions suggested by the reviewers, which we believe have greatly contributed to improving the document. In addition to the issues pointed out by the reviewers, we have applied minor corrections to issues that we had missed previously and minor changes that we believe improve the manuscript. These changes are summarize below in the section “other corrections and additions”. Thus, we provide a revised version of the manuscript with tracked changes. In addition, we included below a point-by-point response to the reviewer’s concerns. To facilitate review, the amendments to the manuscript are highlighted with comments indicating the reviewer comment that they refer to. Importantly, in all the responses below, the lines indicating the amended parts of the revised manuscript refer to the version with “tracked changes”.

Comments from Reviewer #1

This study combines elegantly many different analyses methods from the fields of network neuroscience and computational neuroscience. I congratulate the authors on this fine well-written work. I find this modeling study interesting with dense results that identify a potential mechanism to explain (some parts of) post-stroke recovery. Find some major and minor comments below.

We would like to thank Reviewer #1 for the insightful concerns and encouraging comments on our work. Thus, we carefully addressed all the points expressed below. To facilitate the review of the revised manuscript, all the changes relative to each comment are highlighted in reference to the reviewer's comment they refer to.

Comment 1: *Is there post-lesion an increase in excitation or loss in excitation? I find contradictory statements in the first and last sentence of the second paragraph on the second page (lines 71-82).*

Response: We thank the reviewer for pointing out this issue. In fact, we argue that there is an acute decrease in the levels of incoming excitation to cortical areas that are connected to the lesion. Since most long-range connections in the cortex are excitatory, this would naturally follow the loss of cortical mass following a focal lesion. This is similar to the sensory deprivation protocols commonly used in E-I homeostasis studies (see, for example, Ma et al 2019 Neuron). Therefore, we emphasize the difference between “level of incoming excitation”, which is external to neural populations, and “excitability” which is a property of neural masses, in our model controlled by plasticity of local inhibition. In addition, there is also the issue of timescales, where the decrease in levels of incoming excitation is acute

(immediately post-stroke) and changes in excitability are persistent and chronic (months post-stroke). However, we agree with the concern of the reviewer that this might not be adequately explained in the manuscript, and have thus rephrased the mentioned paragraphs to emphasize the aforementioned differences (Lines 102-114).

Comment 2: *Why did the authors choose AAL parcellation? It seems like an outdated choice.*

Response: We thank the reviewer for raising this methodological point. While it is true that AAL has been shown to underperform when assessed through functional correlation homogeneities see for example Gordon et al 2014 Cerebral Cortex, this is not the case in modeling paradigms where multimodal neuroimaging is concerned. This has been for example demonstrated in a recent work where models based on the AAL parcellations outperformed many of the functionally based atlases (such as the well-regarded Schaefer parcellations) in terms of Inter-Class Correlation - a measure assessing the reliability and sensitivity in terms of within and between subject variability (Domhof et al 2022 Neuroimage). So, given that jury is still out as to what the adequate parcellation should be when considering multimodal approaches such as diffusion and functional MRI, we decided to choose a well-established parcellation shown to perform well in various modeling applications (see Deco et al. 2019 PNAS, Deco et al. 2018 Curr. Biol., Kringelbach et al. 2020, PNAS).

Comment 3: *Why not take the same subjects from the HCP database for the structural and functional matrices?*

Response: We thank the reviewer for addressing this relevant question. In this paper, for obtaining the normative structural connectome, we have used a special subset of 32 subjects available in the HCP database. The main reason for using only such a subsample is that the 32 subjects were recorded using a specially-designed MRI scanner with more powerful gradients than conventional scanners, designed specifically for diffusion imaging. Therefore, we have opted to use this subset of subjects with optimized data quality, together with a state-of-the-art pipeline described in Horn et al 2019 Neuroimage.

Regarding the effects on modeling results, we decided for a normative connectome to avoid the noise in individualized dMRI connectivity. Furthermore,as shown in Domhof et al 2022 Neuroimage, using individualized structural connectivity for modeling is not the most critical factor in improving the within-subject reliability of computational models. Therefore, we argue that using a normative connectome, even if only from 32 subjects, is not a detrimental factor in our results and can aid in the mitigation of noisy connectivity estimates that can be obtained at the individual level. In addition, since in this project we were not focused on personalized models but on the underlying principles of cortical connectivity and its emergent features that might be disrupted following a lesion, we believe that our decision does not have a significant impact on our main conclusions.

Comment 4: *And why not perform even an individual-level fitting here? Since one can assume that the SC-FC coupling should in the individual case be even higher.*

Response: Since our focus was on investigating underlying principles of E-I homeostasis in stroke recovery and its effect at different scales (i.e. local excitability and global emergent properties of FC), we believe that performing an individual level fit to our participants, albeit relevant, would be out of scope of this paper. In addition, regarding the issue of FC-SC coupling, it is indeed likely that it would be higher in the individual level. However, we expect that the main effect observed in our simulations (acute decrease and chronic recovery) would still hold on an individual level, therefore not affecting our conclusions.

On the issue of computation, because we have to ensure that local inhibitory weights reach a steady state before recording activity, we are required to generate hours of simulated activity with a time resolution of 0.2ms. Therefore, performing an entire parameter space run for each of the subjects, with individualized SC matrices, would require several months, even with the use of high-performance computing and optimized code.

On another note, we have performed individual-level fitting with the data that we have at the moment, i.e. comparing the simulations generated from the normative connectome with FC and FCD at a subject level, and we found a high density of individualized optimal points around the optimal region obtained for the group level metrics (Fig. R1). That said, we have included these results in the supplementary figures (Fig. S7) and briefly comment them in the main text (lines 502-505).

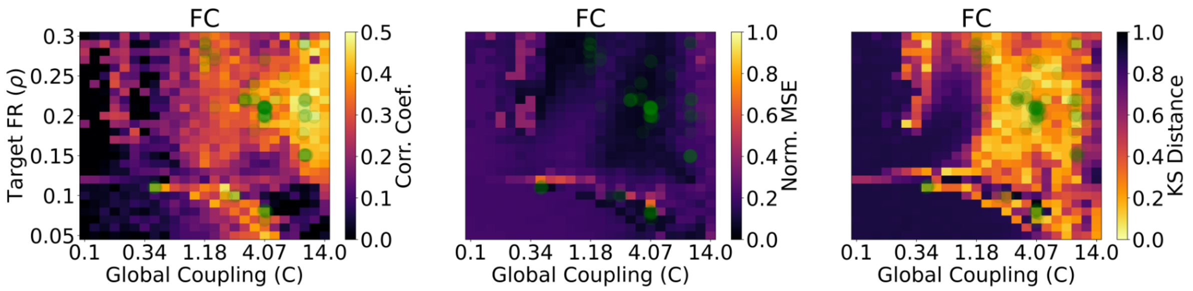

Figure R1 (Fig. S7) - Individual-Level model optimization. The colormaps represent the model's performance compared against averaged FC and the subject aggregated FCD distribution. Green dots represent the optimal point for each subject, obtained when comparing model results against subject specific FC and FCD. Note that most points concentrate around the optimal region chosen as the working point of our model.

Comment 5: *Where did the authors explain how they obtain the tract lengths?*

Response: We thank the reviewer for pointing out this missing aspect in our manuscript. The tract lengths were based on the lengths of the fibers connecting two given parcellated regions and were obtained by computing the three-dimensional distance across all the voxels that the fiber passes through, similar to the method used in Castaldo and Cabral 2022 Commun Phys. We have thus updated our methods section to include this information (lines 193-195).

Comment 6: *Please explain all variables in Eq. (1): C_EI,i what is that? What is N? what is P? what is i?*

Response: We thank the reviewer for pointing out this issue. We have updated our methods section to include a clear explanation of each of the mentioned variables as well as their particular role in node dynamics, particularly for *P.* (lines 225-230, 236-239 and 252-253).

Comment 7: *What is the F-I curve?*

Response: We thank the reviewer for the useful feedback and agree that the use of F-I curve (frequency-current curve) is not best applied in this context. In the revised manuscript, we have therefore changed "F-I curve" to "activation function" and emphasize that this function describes the response of a neural mass to external input. (lines 236-239)

Comment 8: *The described mechanism of E-I homeostasis sounds similar to the mechanism of feedback inhibition control, which is used often to control for over-excitation in modelling studies. Could the authors please comment on the difference between their proposed mechanism and FIC? (e.g. Deco et al, 2014, Deco, G., Ponce-Alvarez, A., Hagmann, P., Romani, G. L., Mantini, D., & Corbetta, M. (2014). How local excitation–inhibition ratio impacts the whole brain dynamics. Journal of Neuroscience, 34(23), 7886-7898.)*

Response: While the implementation of the model is slightly different, the mechanism is essentially the same, based on the adaptation of local inhibition to maintain excitatory activity at a certain target level. Both mechanisms are based on the principles of synaptic scaling (see Turrigiano 2011 Ann Rev Neurosci), which has been shown to occur, among other mechanisms, in the incoming inhibitory synapses to pyramidal neurons. We follow the implementation derived in Vogel 2011 Science which has since been used in large-scale models (see Hellyer 2016 Neuroimage and Abeysuriya 2018 Plos CB). One of the differences is that FIC works with absolute firing rates and, therefore, it commonly uses an empirical estimation of the resting firing rate of cortical networks as the target. In our case, we have used a more abstract notion of activity, interpreted in Abeysuriya et al 2018 as normalized firing rates. Therefore, since the target firing rate (ρ) is also abstract, we fit the models over that parameter as well. To make the relationship between the mechanism we used and FIC more clear, we have updated the Limitations section (4.5) in our manuscript to include a short comparison between the two (lines 1211-1218).

Comment 9: *Why did the authors choose logarithmically spaced values for C?*

Response: One of the interesting properties of models with local E-I homeostasis is that they tolerate a wider range of values of global coupling without reaching saturation in local dynamics. Without homeostasis, we need to be confined to a particular optimal range of *C* to ensure meaningful dynamics, since the activation function of neural masses is sigmoidal and

stronger inputs lead to saturation. Therefore, we wanted to explore a greater range of global coupling than what would be done otherwise. Since it was not clear under which magnitude of coupling the model would still operate reasonably, we decided on logarithmically spaced values of global coupling. To address the reviewer's concern, we have performed more simulations with linearly spaced values around the chosen working point to emphasize that the fit does not change significantly (Fig. R2).

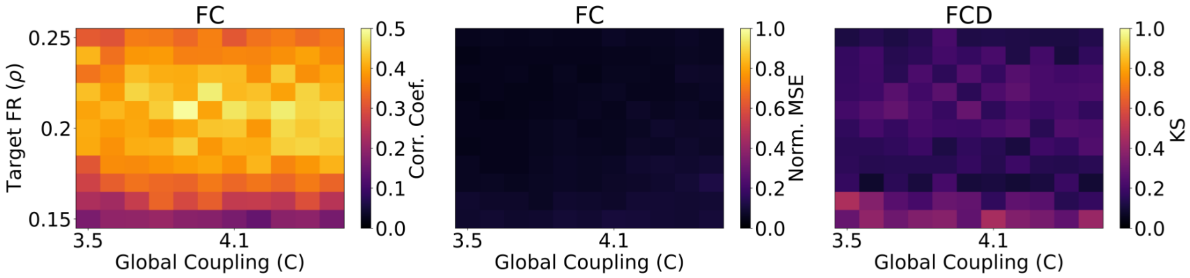

Figure R2 - Model optimization for linearly spaced values of global coupling (C). We show results of the optimization for values of C in the vicinity of the chosen working point, with a spacing of 0.1. Not that, within the range of values presented in this figure, model performance does not vary significantly.

Comment 10: *Line 270: typo matrices*

Response: We thank reviewer 1 for spotting the typo, which has been corrected in the revised version of the manuscript.

Comment 11: *Please explain abbreviations in Fig. 1 in the legend*.

Response: We thank reviewer #1 for the suggestion. To improve the clarity of our manuscript, we have included explanations for the abbreviations in Fig.1.

Comment 12: *Another modelling aspect the authors could try to relate to empirical stroke patient finding would be the lengths of the stabilization period in their different virtual lesion simulations. If the authors would have any interpretation about what time frame we are looking at in the simulations and compare the relative stabilization period lengths with literature about which regional lesions are known to have a faster recovery relative to other regions. This could be an interesting additional aspect that the authors could check.*

Response: We thank the reviewer for the relevant suggestion. Theoretically, under the framework of local E-I homeostasis, it's trivial that lesions in nodes with high strength (sum of structural connectivity) will require a longer stabilization period since they entail stronger changes in the levels of incoming excitation to a larger number of other nodes in the network. Given the homeostatic mechanism implemented in our model, the way local inhibitory weights change after a perturbation is similar to exponential decay (see Fig. S2). Therefore, a stronger acute drop in incoming excitation will require a longer time to return

excitatory activity to the target firing rate. We agree that this is an interesting property to link to empirical results, although we should be conservative in attributing causality of longer recovery times in stroke patients to E-I homeostasis solely. However, we have found in the literature that there is indeed a high heterogeneity in the extent of spontaneous recovery in the first three months post-stroke related to lesion size and location, which is in line with our work, since lesions in strongly connected nodes require longer times for E-I homeostasis to achieve its target (Fig. R3). Therefore, we added a paragraph in the discussion to delve into this issue (lines 1059-1074) and a new supplementary figure (Fig. S14) to illustrate this concept. In addition, we rephrased the title of section 4.3 to include not only the possible impairments, but also its connection to recovery times.

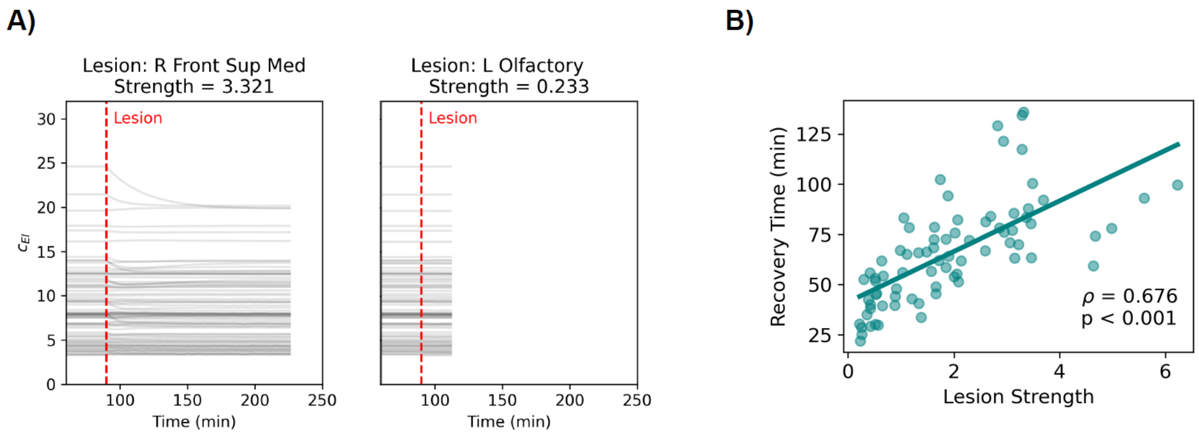

Figure R3 (Fig. S14) - Post lesion adaptation time is dependent on lesion strength. A) Example of the evolution of local inhibitory weights c_{EI} after lesion in a high strength node (right superior frontal gyrus, medial orbital) and a low strength node (left olfactory cortex). B) Correlation between lesion strength and time required for c_{EI} stabilization after lesion. Lesions in nodes with strong structural connections require longer adaptation times for the restoration of local E-I balance.

Comment 13: *Though the authors mention "multiscale" in the title, they do not use it in the main text except for pointing towards another study. I suggest to remove it from the title or explain more directly in the manuscript how the word is to be understood in this study.*

Response: We appreciate the fact that the reviewer brought forward this issue. Modularity, for example, is considered by us to be a global scale (i.e. macroscale) property of FC. It is a property that makes sense when considering the whole network and it is not reducible to its constituting elements (single nodes), reflecting the structure of interactions between them instead. A similar argument can be applied to the SC-FC coupling, which is related to how much the functional networks reflect the underlying structure, being, in our opinion, a global scale property as well. On the other hand, even though the plasticity mechanisms responsible for E-I homeostasis happen on the level of the synapse, our model uses a mesoscale abstraction of such mechanisms, at the neural-mass level. Thus, changes in excitability in our model happen on a local scale (i.e. mesoscale) and can be understood at the level of each of the single units constituting the network. Using the framework introduced in Betzel and Bassett 2016 Neuroimage (see Fig. 1), our processes would be then

distributed along the spatial and topological scales, with E-I homeostasis at the population level on the spatial scale and local level on the topological scale, and modularity in the global level of the topological scale. That considered, we use the term multiscale to encompass these two scales and emphasize the fact that a mesoscale mechanism, E-I homeostasis, has effects that extend beyond the regulation of mesoscale dynamics into the re-emergence of global properties of FC such as modularity. While it is true that we do not use the word "multiscale" regularly in our manuscript, we do mention that we attempt to study the effects of focal lesions at both a local (mesoscale) and global (macroscale) level as in, for example, the last paragraph of the introduction:

"Therefore, we hypothesize that E-I homeostasis not only plays an important role in the maintenance of E-I balance at the mesoscale but also in the recovery of macroscale properties of FC (i.e. modularity and small-worldness)."

That said, to address the concerns of both reviewers that the reason why we consider our exploration to be multiscale is not clear, we have reformulated our introduction and discussion sections to emphasize the aforementioned points more strongly and to explicitly employ "multiscale" where the term is due (lines 156-168, 964-969, 1266-1269).

Comments from Reviewer #2

Authors explore the effects of excitatory-inhibitory homeostasis in a whole-brain model based on the connectome and the WC model. The results are not directly linked to empirical data, but the computational exploration is nevertheless technically very sound and solid and the paper is very well written.

The framing of the problem, and the link with the empirical studies is solid.

However, I still have some issues that I would like to see being resolved before I suggest a publication of the paper.

We would like to thank Reviewer #2 for the insightful and challenging concerns and for the positive comments on our work. Thus, we carefully addressed all the concerns expressed below. To facilitate the review of the revised manuscript, all the changes relative to each comment are highlighted in reference to the reviewer's comment they refer to.

Comment 1: *I don't see a justification for using the adjective multiscale in the title.. even though the mechanisms of the E/I homeostasis are on microscale, still the WC model is quite phenomenological in order to call the mechanisms multiscale.*

Response: We thank the reviewer for pointing out this issue. Shortly, we use the adjective multiscale not for the modeling approach per se, but for the way we explore the effects of focal lesions. Modularity is, in our view, a macroscale property of cortical connectivity, since it cannot be understood at the node level. Conversely, the changes in excitability are instead considered to be mesoscale, since they relate to individual nodes. Therefore, our approach is multiscale, since we attempt to bridge the mesoscale E-I balance with the emergence of macroscale properties. For more detail, please refer to the response to Comment 13 from reviewer 1.

Nonetheless, we acknowledge the concern that such interaction between scales may not be clear in the current iteration of the manuscript, and have updated our introduction and discussion sections to more clearly emphasize the multiscale aspect of our work (lines 156-168, 964-969, 1266-1269).

Comment 2: *why the graph theory metrics are applied on thresholded and binarized matrices, when the same metrics are available for weighted matrices in the brain connectivity toolbox for example?*

Response: We thank the reviewer for this relevant question. The main reason for this particular implementation is to follow the methodology previously applied to empirical data from stroke patients by Siegel et al 2018 Cortex. Being, to our knowledge, the main paper

relating stroke to changes in modularity, we believe that it would be the best option to compare our model results with empirical findings. Nonetheless, we have performed the same analysis in the weighted connectivity matrices, as described in the same paper, which we have added to the supplementary material as Fig. S5d. Importantly, the results remain quantitatively very similar (Fig. R4).

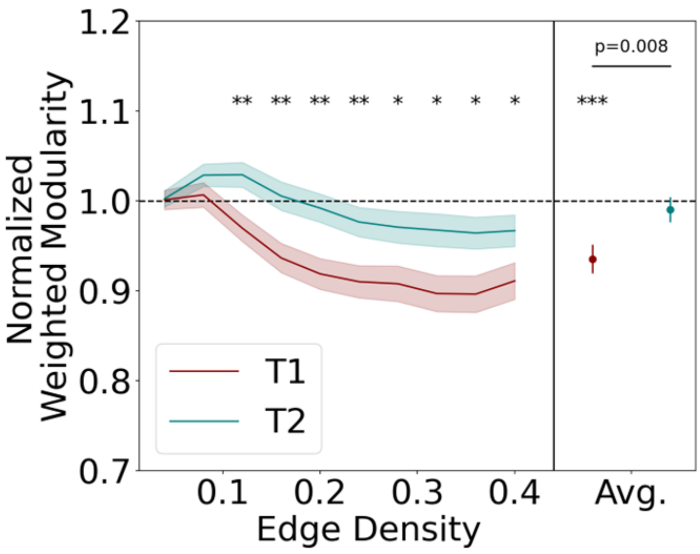

Figure R4 (Fig. S5d) - Weighted modularity at T1 and T2, normalized to values at T0, for a range of edge density thresholds. On the right, we plot the average across density thresholds for T1 and T2. In qualitative terms, using a weighted version of the modularity algorithm presented in Siegel et al 2018 Cortex does not alter our results significantly.

Comment 3: *the optimal conduction speed of 39m/s is unreasonably too high for cortico-cortical axons… for example, it is almost 10 times higher than the value obtained from the largest database of cortico-cortical evoked potential (Le Marechal et al Brain 2021), and the same mean value of ~3.3m/s for example was shown to be needed to reproduce some the major frequency-dependent activation patterns in Petkoski et al Netw Neurosci 2022. I'm aware that some other simplifications (e.g. setting 40Hz in every region) in the model might be responsible for such a high value, but I expect the issue of the propagation velocity to be at least properly discussed.*

Response: We thank the reviewer for raising this relevant issue. We do agree that, on first inspection, such a value seems unreasonably high. Indeed, the fact that we base our model on gamma oscillations might require faster delays to preserve adequate phase relations (see, for example, Cabral and Castaldo 2022 Commun Phys., where delays with the same magnitude were found). While this can partially explain the short delays, as we comment in the revised manuscript, we believe that these values might still be within reasonable bounds. More specifically, we use long-range white matter tracts (which are heavily myelinated), as the structural connectivity constraining our model. While the axon conduction velocity in unmyelinated axons is indeed in the range from 0.5 to 10m/s, myelinated fibers, depending on the level of myelination and their diameter, can conduct signals at velocities up to 150

m/s. (see, for example https://www.ncbi.nlm.nih.gov/books/NBK10921/ and https://www.nature.com/scitable/topicpage/myelin-a-specialized-membrane-for-cell-communication-14367205/). In addition, studies have shown that the distance between Ranvier Nodes in myelinated fibers can increase significantly the conduction velocity, up to a factor of 4, from around 5m/s to 20m/s (https://jnnp.bmj.com/content/jnnp/40/8/769.full.pdf).

Furthermore, recent attempts at quantifying the conduction velocity of long-range white matter tracts using microstructural metrics extracted from MRI signals provide an approximation of conduction velocity at around 14 m/s. While still considerably slower than the velocity we have implemented, it is still significantly faster than the suggested 3 m/s (see https://www.ncbi.nlm.nih.gov/pmc/articles/PMC8448685/, using the method validated by https://www.sciencedirect.com/science/article/pii/S1053811919307773). In our view, given the sometimes contradictory results, it is still unclear how fast signal conduction should be in large-scale models to accurately represent brain activity and thus we believe that our fast delays do not present a significant caveat to our results. In addition, it is important to mention that we are defining our conduction delays based on fiber tract length, as opposed to euclidean distances between ROI centroids, which leads to longer fiber lengths, particularly in interhemispheric connections, that in turn may require faster conduction velocities.

Unfortunately, we were unable to find the Le Marcehal reference mentioned by the reviewer and thus cannot comment on it. Regarding the other reference, Petkoski et al Net Neurosci (https://direct.mit.edu/netn/article/6/3/722/109167/Normalizing-the-brain-connectome-for-communication) we could not find any reference to the extracted conduction velocities. However, in another recent work of some of the authors, conduction velocities were found to be greatly heterogeneous, as opposed to our approach, where they are considered homogeneous instead (https://pubmed.ncbi.nlm.nih.gov/36241383/). This is indeed a caveat of our study, which we have added to the commentary in the revised manuscript.

In conclusion, we agree that we should interpret the optimal conduction velocity in our model with more care in the manuscript, and have thus extended the respective part in the results section to encompass a more complete commentary on the value we obtained. (lines 476-480)

Comment 4: *l499 is there any indication that it is more new connections, or strengthening of the old one? It should be checked in the model, but also linked to what is known in the literature.. Similar to this it should be discussed that structural rewiring is another phenomena that might shape the changes in FC due to stroke.. for example it is known that there is a contralateral increase of connectivity in stroke (e.g. see the review by Nudo et al 2013), and this was then used to model FC changes of calcium activity in stroke in mice in Allegra et al Front Sys Neurosci 2020.. also in the same study the changes of neuromodulation were used as mechanism for modelling the pericital local changes in the dynamics..*

Response: We thank reviewer 2 for these relevant suggestions. Prompted by these comments, we performed some extra analysis to be able to understand this question better and added a new supplementary figure, now S8, to summarize our results. There, we simply

correlated the variation in FC between different points in time in our simulations with either SC or healthy FC(T0) (Fig. R5). The main conclusions are:

- Generally, across lesions, there is a high negative correlation between $\Delta FC_{1\text{-}0}$ and $\Delta FC_{2\text{-}1}$, showing that the improvements from the acute to the chronic period tend to counteract the deficits, showing that FC is indeed being recovered from T1 to T2. This is in line with recent findings in the literature (Olafson et al 2021 Neuroimage), which we reference in the revised manuscript.
- Regarding what we call reorganization, meaning the differences between T2 (chronic period) and T0 (healthy brain) which are found after recovery, we found that they are not correlated with structural connectivity but they seem to be negatively correlated, for most lesions, with healthy FC. This means that connections that increase between T0 and T2 are the ones that are weakest at T0. In general terms, this can be understood as formation of new functional pathways, instead of strengthening of previous ones. This effect survives even if we account for the fact that weaker connections have more potential to increase, by normalizing changes between T0 and T2 to the maximum possible change (i.e. 1-FC(T0)).

In light of the above, we have updated the results and discussion sections of our manuscript to make it more clear that the reorganization we observe in the model is better interpreted as the formation of new functional connections (lines 560-568, 1008-1017).

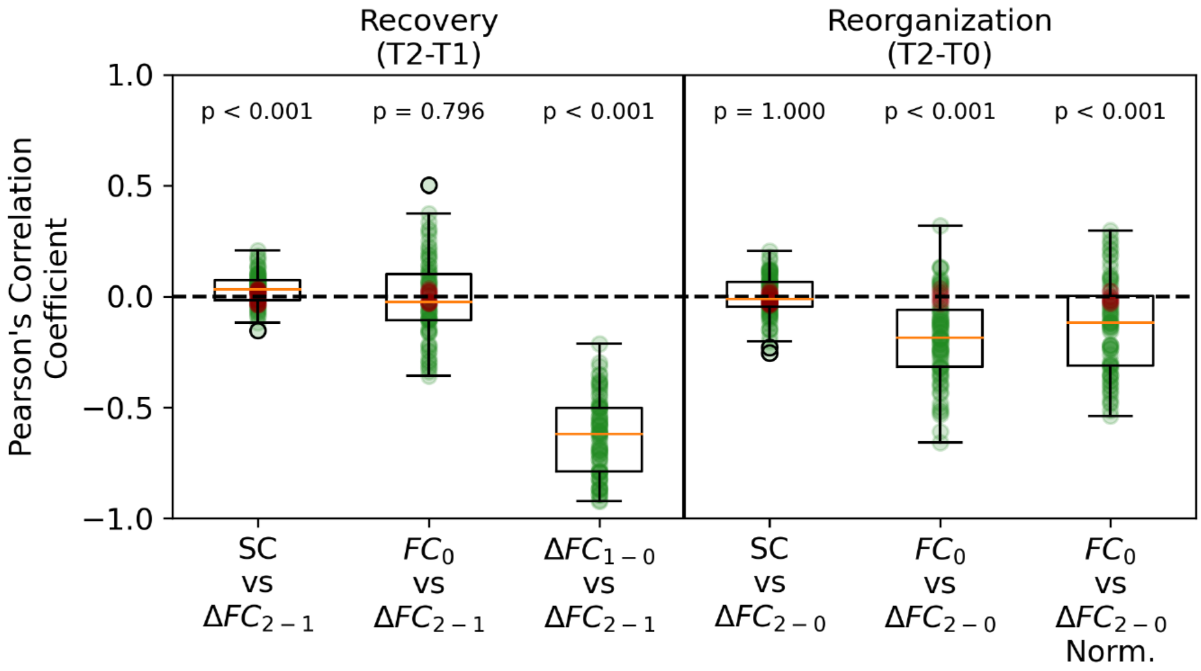

Figure R5 (Fig. S8) - Functional connectivity recovery and reorganization Correlation between recovery and reorganization of FC across lesions. We classify differences between T1 and T2 as recovery, since they are related to the recovery of acute FC deficits, and differences between T2 and T0 as reorganization, since they quantify the reorganization of FC connections that is required for recovery. From left to right, we show the correlation across lesions between SC and ΔFC(T2-T1) FC(0) and ΔFC(T2-T1), ΔFC(T2-T1) and ΔFC(T1-T0), SC and ΔFC(T2-T0), FC(T0) and ΔFC(T2-T0), FC(T0 and ΔFC(T2-T0) normalized to the maximum possible change, i.e ΔFC(T2-T1)/FC(T0). Dots represent the

correlation between each of the combinations of measures on an individual lesion level. Dot color indicates the significance of the correlation, with green representing FDR corrected p-value lower than 0.05. Boxplots represent the distribution across lesions. P values above each boxplot represent the result of a Wilcoxon ranked sum test. Note that recovery is strongly negatively correlated with acute deficit in FC (third boxplot), indicating that deficits are being appropriately corrected from T1 to T2 In addition, reorganization is generally negatively correlated with healthy FC(T0) (fifth boxplot), meaning that reorganization is underlined by increases in the weakest healthy functional connections, which can be conceptualized as the formation of new functional connections. The significance of this result is maintained when normalizing for maximum possible increase in FC (sixth boxplot).

Regarding the second part of the comment, referring to structural rewiring, we agree that it is indeed a relevant mechanism of stroke recovery. We do mention, in the discussion, recent results (see Rocha et al 2022 Nat Comm) indicating that structural plasticity might be essential for the recovery of global dynamics (such as criticality), which, according to our results, cannot occur only through local E-I homeostasis. That said, and since the literature points out that structural rewiring is a relevant aspect of functional recovery, we have updated the discussion of our manuscript to include some short discussion on this issue (lines 1227-1233), suggesting that further modeling endeavors could include both forms of plasticity. However, since we have not implemented any form of structural plasticity in our model, we believe that discussing this question in more detail is beyond the scope of our work.

Comment 5: *it is worth at least mentioning that effective connectivity has been shown to be better predictive for stroke than FC, Adhikari et al Brain 2021.*

Response: Since we wanted to emphasize the re-emergence of macroscale properties of FC, such as modularity, from mesoscale regulation of E-I balance, we focused on previous stroke research that discusses these macroscopic network measures. We do agree that the research on EC is a relevant emerging field, as evident from the paper suggested by the reviewer. Nonetheless, the suggested paper focuses on feeding EC to a classifier to distinguish stroke patients from healthy subjects, an approach that, although very relevant, goes beyond the mechanistic approach we tried to follow in our work. That said, we have updated our discussion to include EC and its potential further applications in the modeling of stroke. (lines 1244-1246).

Comment 6: *I applaud the authors for their focus on FCD, even though as they admit there is still not much empirical results for this case.. maybe they should also mention that FCD is becoming proven as more informative than FC for ageing, cognitive performance, etc.. However, they seem to have omitted a very recent work that uses FCD based metric, Kim et al Nat Comm 2022.*

Response: We thank reviewer 2 for pointing out this issue. Indeed, we agree that our discussion on FCD was lacking depth in the original version of the manuscript and have thus expanded our introduction (lines 92-101) to include some considerations on the relationship between FCD and higher-order cognition and behavior. Furthermore, we added some discussion on recent work from Anna Bonkhoff, the most recent results we found discussing dynamical functional connectivity in stroke patients (lines 1038-1058). Unfortunately, we were not able to find the paper mentioned by the reviewer, but we further comment on a recent study by Favaretto et al in Nat Comm 2022 that focused on cortico-subcortical dynamics, but where the results indicate that, at least in severely impaired stroke patients, dynamics seem to be less flexible, in line with our speculations

Comment 7: *when discussing Fig. 5 A, authors should be clearer that the Euclidean distance is relevant only because the connectivity decreases with the distance, and the topology is the only determinant of their results.*

Response: We thank reviewer 2 for pointing this out. We do agree with the comment, as mentioned in the manuscript ("*We chose to explain these variations as an exponential function of distance given the exponential dependence found between structural connectivity and distance in the cortex (95) and the fact that areas more strongly connected to the lesion would experience the strongest loss in excitation"*). Furthermore, fitting the changes in local inhibitory weights with an exponential function further strengthens this claim, since connectivity is known to decrease exponentially with distance. Therefore, our aim was to show that the distance dependence of changes in excitability found previously in rodent research is trivial under the framework of E-I homeostasis. That said, we added an extra remark to the respective section in our manuscript to emphasize this effect and clarify that we believe this distance rule is the driver of the observed changes (lines 771-774).

Comment 8: *when discussing the importance of the graph theory metrics and topology for the stroke impact, it is maybe interesting to make a link that the same is the case for number of lesions to control seizure propagation in epilepsy, which is highly correlated with the graph theory properties of the node, Olmi et al Plos CB 2019.*

Response: We thank reviewer 2 for the suggestion of this reference, which does strengthen our point about the relevance of the graph properties of lesioned areas for the network-level alterations. We have thus added a short comment on the paper and how its conclusions tie to our own discussion. (lines 1102-1109)

Comment 9: *as for the limitations, it is worth mentioning the fact that they assume same strength of homeostasis at every brain region, while as I mentioned in the previous comments, it is expected regions strongly connected to the stroke region, and especially the same contralateral region, to be more affected by structural alterations, which don't exclude the E/I homeostasis.*

Response: We thank the reviewer for this suggestion. This could be indeed a limitation of our study and possible inclusion in further modeling studies. To our knowledge, deficits in EI homeostasis have not been explored in the stroke brain, as we emphasize in the discussion (see Section 4.3), so it is difficult to discuss this with empirical backing. Therefore, in the revised manuscript, we suggest it as a line of research for the future (lines 1112-1125) referring that deficits in E-I homeostasis might be found in areas that are either in the vicinity or strongly connected to the lesioned area. Importantly, as our results show, these are generally the areas that require stronger homeostatic adaptation, thus raising the importance of studying the spatial distribution of E-I homeostasis impairments.

Comment 10: *l681 using further as a verb doesn't sound right.*

Response: While the word further can be used as a verb, meaning to help forward *(https://www.merriam-webster.com/dictionary/further),* we agree that it is not the best word for the context. Thus, we have changed it to "extend" in the revised manuscript to improve clarity.

Other corrections and additions

- Added a sentence to the introduction to emphasize some post-stroke cognitive deficits in the introduction (Lines 60-63).
- Rephrased sentence regarding the results on modularity to improve clarity (Lines 591-595)
- Rephrased sentence regarding cortical reorganization in the results section to improve clarity (Lines 613-615)
- Rephrased sentence on the excitability results to improve clarity (Lines 795-799)
- Improved sentence in the discussion to elucidate some of the possible causes of the discussed side effects of stroke, besides maladaptive plasticity (Lines 985-986)
- Rephrased a sentence in the discussion of possible impairments in E-I homeostasis (Lines 1090-1091)
- Rephrased section in the discussion of stroke side-effects improve clarity (Lines 1169-1170)
- Rephrased sentence on the limitations of our implementation of E-I homeostasis (Lines 1210-1211)
- Added the following sentence to close the limitations section: "Therefore, despite the caveats, our biologically constrained model succeeds in answering stroke-specific physiological benchmarks leading to a new set of predictions that can be validated with current experimental methods." (Lines 1249-1252)

References:

Abeysuryia 2018 Plos CB -
https://journals.plos.org/ploscompbiol/article?id=10.1371/journal.pcbi.1006007

Betzel and Bassett 2016 Neuroimage -
https://www.sciencedirect.com/science/article/pii/S1053811916306152

Brill et al 1977 Journal of Neurology, Neurosurgery, and Psychiatry -
https://pubmed.ncbi.nlm.nih.gov/925697/

Castaldo and Cabral 2022 Commun Phys - https://www.nature.com/articles/s42005-022-00950-y

Deco et al. 2018 Curr. Biol. -
https://www.cell.com/current-biology/pdfExtended/S0960-9822(18)31045-5

Deco et al. 2019 PNAS - https://www.pnas.org/doi/10.1073/pnas.1905534116

Domhof et al. 2022 - https://www.sciencedirect.com/science/article/pii/S1053811922004402

Drakesmith et al 2016 Neuroimage -
https://www.sciencedirect.com/science/article/pii/S2213158216301607

Favaretto et al in Nat Comm 2022 - https://www.nature.com/articles/s41467-022-32304-1

Gordon et al 2014 Cerebral Cortex - https://pubmed.ncbi.nlm.nih.gov/25316338/

Hellyer 2016 Neuroimage - https://pubmed.ncbi.nlm.nih.gov/26348562/

Horn et al 2019 Neuroimage - https://pubmed.ncbi.nlm.nih.gov/30179717/

Kringelbach et al. 2020, PNAS - https://www.pnas.org/doi/10.1073/pnas.1921475117

Ma et al 2019 Neuron - https://www.cell.com/neuron/pdf/S0896-6273(19)30737-8.pdf

Mancini et al 2021 Brain Struct Funct - https://link.springer.com/article/10.1007/s00429-021-02358-w

Olafson et al 2021 Neuroimage -
https://www.sciencedirect.com/science/article/pii/S1053811921009150

Purves et al 2001 Neuroscience - https://www.ncbi.nlm.nih.gov/books/NBK10921/

Rocha et al 2022 Nat Comm - https://www.nature.com/articles/s41467-022-30892-6

Siegel et al 2018 Cortex - https://www.ncbi.nlm.nih.gov/pmc/articles/PMC6527102/

Susuki 2010 Nature Education -
https://www.nature.com/scitable/topicpage/myelin-a-specialized-membrane-for-cell-communication-14367205/

Turrigiano 2011 Ann Rev Neurosci - https://pubmed.ncbi.nlm.nih.gov/21438687/

Vogels 2011 Science - https://pubmed.ncbi.nlm.nih.gov/22075724/
