# Supplementary material for: Multiscale effects of excitatory-inhibitory homeostasis in lesioned cortical networks: A computational study
Source: PLoS Comput Biol. 2023 Jul 7;19(7):e1011279. doi: 10.1371/journal.pcbi.1011279 (PMC10355437; doi:10.1371/journal.pcbi.1011279)
Supplement: Attachment — Submitted filename: Reviewers Comments 2.pdf [file pcbi.1011279.s017.pdf]

Dear Editors,

We thank you for the opportunity to resubmit a revised version of the manuscript for publication in the PLOS Computational Biology journal. We have been able to update our manuscript to include the suggested revisions. Thus, we provide a revised version of the manuscript with tracked changes.

Comments from Reviewer #1

The authors have addressed my comments to my satisfaction. I am impressed with the addition of new results based on my comments and thank the authors for their additional work, which in my opinion strengthened the manuscript.

We would like to thank Reviewer #1 for the encouraging comments on our manuscript. We agree that the changes made as a response to the suggestions of the reviewer have improved our manuscript and broadened the reach of our results.

Comments from Reviewer #2

The authors have seriously taken all of my comments, and they have in-depth addressed all of them.

The only remaining issue is remaining the conduction velocity. Most of the cited references by the authors in their response, do not correspond to cortico-cortical neurons, but also to the spinal cord, where those high velocities are recorded. see for example http://www.scholarpedia.org/article/Axonal_conduction_delays or as I mentioned, I still consider the Lemarcehal et al. Brain paper as an actual gold standard, since it does measure the velocity in human over the whole cortex. This time I'm also sending the link for the paper and it's doi: https://pubmed.ncbi.nlm.nih.gov/35416942/, doi: 10.1093/brain/awab362.

Referring to purely computational studies, where the velocity is just fitted to get a good correspondence (as in the mentioned Cabral paper) is a weak argument, though I do understand that due to different approximation sometimes models might require non-realistic values for the conduction velocity. And that is still acceptable (for me at least) for as long as it is properly discussed.

We thank the reviewer for the positive feedback and insightful comments. In particular, we appreciate that the reviewer pointed out that our arguments were mostly based on results obtained from corticospinal fibers and that a gold standard was provided. We have thus updated the Discussion section of our manuscript (Section 4.5: Limitations) to include a paragraph dedicated to this issue, addressing in more detail the discrepancy between the delays used in our work and the empirical results. For this reason, we have decided to remove the short discussion on mean delays from the Results section and explore the question more in-depth in the Discussion (Lines 1161-1184).
